# Entropy-Regularized Hierarchical MARL for Resilient Moving Target Defense in Cyber–Physical Systems

**DOI:** 10.3390/e28070775

**Published:** 2026-07-08

**Authors:** Atef Gharbi, Ahmad Alshammari, Nadhir Ben Halima

**Affiliations:** 1 Department of Information Systems, Faculty of Computing and Information Technology, Northern Border University, Rafha 91911, Saudi Arabia; 2 Department of Computer Sciences, Faculty of Computing and Information Technology, Northern Border University, Rafha 91911, Saudi Arabia; ahmad.almkhaidsh@nbu.edu.sa; 3 Department of Information Technology, Community College of Qatar, Doha 7344, Qatar; nadhir.benhalima@ccq.edu.qa

**Keywords:** multi-agent reinforcement learning, moving target defense, cyber–physical systems, hierarchical control, partially observable stochastic games, k-winner-take-all, centralized training decentralized execution, entropy

## Abstract

Cyber–Physical Systems (CPS), including smart grids and industrial control networks, must maintain secure and stable operations despite increasingly adaptive cyber threats. Existing moving target defense (MTD) approaches often rely on fixed reconfiguration strategies or flat learning architectures that fail to scale and do not explicitly ensure operational resilience under real-time constraints. This study proposes a resilience-oriented hierarchical multi-agent reinforcement learning (MARL) framework for adaptive MTD in CPS environments. The attacker–defender interaction is modeled as a partially observable stochastic game, enabling defenders to learn adaptive strategies with incomplete information. The proposed architecture consists of three layers: a strategic MARL layer that optimizes high-level defense parameters, a distributed k-winner-take-all coordination layer for low-latency defender selection, and a robust execution layer based on sliding-mode control to preserve physical system stability during reconfiguration. By decoupling strategic adaptation from real-time control, the framework improves scalability and supports resource-aware defense through selective agent activation. Extensive simulations with up to 50 defender agents demonstrate that the proposed approach achieves a defense success rate of 92.4%, reduces the response time by 15% compared with the random MTD, and lowers the energy consumption by 34% on average (up to 52% at *N* = 50) relative to the flat MARL. These results indicate that hierarchical MARL can significantly enhance CPS resilience by enabling adaptive, efficient, and operationally safe defenses against dynamic cyber-attacks. The proposed framework is particularly suitable for edge-enabled CPS environments with strict, real-time, and safety constraints.

## 1. Introduction

Cyber–Physical Systems (CPS), including smart grids and industrial control networks, tightly integrate sensing, communication, and control to support the autonomous and efficient operation of critical infrastructure [1]. However, this deep integration significantly increases the system’s exposure to cyber threats, such as false data injection and coordinated multi-point attacks that simultaneously target cyber and physical components [2,3,4,5]. The heterogeneity of CPS elements, ranging from sensors to programmable logic controllers and edge devices, further complicates the design of security mechanisms that must operate reliably under strict timing, safety, and resource constraints [6,7].

Traditional static security mechanisms are increasingly inadequate in such environments, as attackers can observe system behavior over time and exploit predictable defense patterns [8]. To address this limitation, Moving Target Defense (MTD) has been proposed as a proactive strategy that continuously changes system configurations to increase uncertainty and reduce the attacker’s ability to plan effective intrusions [9,10,11]. Recent advancements have extended MTD beyond network-level defenses to encompass physical-layer adaptation and control-aware reconfiguration in CPS [12,13,14]. Numerous methodologies depend on predefined rules or optimization-based models, which constrain their responsiveness as adversaries evolve and modify their behavior over time [5,15].

In this context, multi-agent reinforcement learning (MARL) has emerged as a prominent tool for adaptive and distributed cyber defense systems. MARL enables multiple agents to acquire coordinated policies through environmental interaction, effectively aligning with decentralized cyber–physical system (CPS) architectures [16,17,18]. Cybersecurity challenges frequently exhibit adversarial dynamics, prompting researchers to employ game-theoretic frameworks, such as partially observable stochastic games, to model these scenarios. These models encapsulate uncertainty and incomplete information in attacker–defender interactions [19,20].

Even with these strengths, the flat MARL architecture has clear limits on how large it can be. As the number of agents increases, the joint action space expands rapidly, making learning more difficult. Simultaneous updates across agents create non-stationary dynamics that render training unstable [21,22]. These issues reduce the suitability of these methods for large-scale CPS deployments.

Hierarchical reinforcement learning addresses these challenges by dividing decision-making into several levels. High-level layers handle strategic planning, whereas lower layers manage execution tasks [23,24]. This structure reduces the learning complexity and improves scalability. However, many existing designs do not provide a tight link between distributed coordination and physically safe execution. In CPS environments, such integration is essential to ensure that adaptive defense actions do not violate physical constraints or destabilize system operations during reconfiguration [25].

From an information-theoretic perspective, adaptive cyber defense can be interpreted as the management of uncertainty in adversarial environments. Entropy offers a direct measure of policy unpredictability, which plays a key role in MTD settings, where attackers can learn and exploit deterministic strategies. Maintaining an appropriate level of entropy enables the system to balance exploration and exploitation while preserving operational stability, thereby increasing the difficulty of attack prediction without introducing unsafe behaviors.

Motivated by these challenges, this study proposes an entropy-regularized hierarchical MARL framework for adaptive Moving Target Defense in cyber–Physical Systems. The proposed design combines strategic learning, distributed coordination, and robust execution in a single architecture. This integration supports scalable and efficient defense while preserving physical safety against dynamic cyber threats.

The main contributions of this study are summarized as follows:

1. A resilience-oriented hierarchical MARL framework that combines strategic adaptation, distributed coordination, and robust execution for the defense of CPS.
2. A game-theoretic formulation of attacker–defender interaction as a partially observable stochastic game, enabling adaptive decision-making under uncertainty.
3. A distributed k-winner-take-all coordination mechanism for efficient and resource-aware defender selection.
4. A physically safe execution layer based on sliding-mode control to ensure stability during dynamic reconfiguration.
5. A comprehensive experimental evaluation demonstrated improvements in the defense success rate, response time, energy efficiency, and scalability.

The remainder of this paper is organized as follows. Section 2 reviews the relevant literature. Section 3 defines the system model and problem formulation. Section 4 details the hierarchical MARL framework (Materials and Methods). Section 5 presents the simulation results. Section 6 discusses the study findings. Finally, Section 7 concludes the study.

## 2. Related Work

The CPS security landscape is evolving rapidly owing to increasingly complex cyber-attacks. We review the literature in three key domains: MTD in CPS, MARL for cybersecurity, and distributed task allocation.

### 2.1. Moving Target Defense in Cyber–Physical Systems

Moving Target Defense (MTD) moves cybersecurity away from fixed protection boundaries toward systems that change their configuration over time [9]. Early studies on cyber–physical systems focused on network-level techniques, such as IP hopping, to interrupt attacker reconnaissance and scanning activities [10]. More recent studies have pushed MTD beyond the network layer, extending it to physical processes and control mechanisms within the system. Kanellopoulos et al. [12] developed a control framework that continuously alters parameters to hinder attacker knowledge acquisition while maintaining stability. Lakshminarayana et al. [13] designed an MTD strategy to detect coordinated attacks on power grids by perturbing branch impedances. Complementary resilient-CPS mechanisms include k-WTA-based distributed selection [26,27,28] and sliding-mode resilient control for maintaining stability under cyber-induced perturbations [29,30]. Topology switching constitutes a further MTD strategy applicable to counter false data injection within distribution networks [14,31]. Although these approaches are grounded in robust theoretical principles, a significant number of them depend on static rules or inflexible optimization methodologies. Consequently, these designs are constrained in their capacity to adapt as adversaries evolve and modify their tactics over time [5,15]. To overcome this limitation, we propose a learning-driven MTD framework that dynamically adjusts its operational behavior in response to evolving attack patterns [32]. Recent secure-control studies have also addressed deception attacks in nonlinear networked CPS through adaptive neural and self-triggered mechanisms, emphasizing the importance of resilient control under adversarial uncertainty and communication constraints [33].

### 2.2. MARL and Game Theory for Cybersecurity

The adversarial nature of cybersecurity naturally aligns with game theory and reinforcement learning. Recent studies have highlighted the growing use of Multi-Agent Reinforcement Learning (MARL) in automated cyber defense [16,17,18]. The attacker–defender interaction is frequently modeled as a Partially Observable Stochastic Game (POSG) [19,20]. However, flat MARL architecture faces significant scalability issues in large-scale cyber–physical system (CPS) networks. This is due to the exponential growth of the action space and the non-stationarity caused by simultaneous learning, as shown in previous studies [21,22]. To mitigate this, hierarchical MARL (HMARL) and CTDE architecture have been proposed [34]. Singh et al. [23] showed that decomposing cyber network defense into strategic and tactical layers can improve learning efficiency. Alqithami et al. [24] integrated adversarial resilience into HMARL. Our framework is tailored to CPS physical constraints by separating high-level MTD parameter selection from robust physical control execution [25].

### 2.3. Distributed Task Allocation and Coordination

In multi-agent CPS defense, high-level strategies must be translated into coordinated agent actions. Distributed task allocation is critical because central coordinators represent vulnerable single points of failure [35]. The k-WTA mechanism is an efficient and competitive network model for resource allocation [26]. Li et al. [27] showed that k-WTA networks can tolerate noise in multi-agent tracking tasks. Auction-based methods often introduce communication delays, which reduce their effectiveness in time-critical scenarios. In contrast, the k-WTA mechanism supports a fast and decentralized role assignment based on locally computed fitness scores [28]. In this study, we used a modified k-WTA scheme to link the strategic MARL and physical control layers. This design supports efficient task allocation under strict timing constraints and varying energy levels across agents [36]. Recent reinforcement-learning-based consensus approaches have further shown that nonlinear multi-agent systems can achieve predefined-time fault-tolerant coordination under dynamic operating conditions [7].

As summarized in Table 1, the comparison shows that most existing CPS defense methods only meet a few of the most important requirements for resilient operations. Rule-based and optimization-driven MTD methods offer stable behavior; however, they have difficulty adapting when attackers learn and change their tactics. Flat MARL methods exhibit adaptive behavior; however, they face limitations in terms of scale and may generate unsafe actions because they lack explicit physical constraints. Hierarchical reinforcement learning has been studied in related areas, but many designs do not link distributed coordination with safe physical execution in a tight and consistent manner. In this study, the proposed framework combines these elements into a single architecture. It combines adaptive strategy learning, decentralized coordination, and robust control execution. This integration supports scalable and safe defenses in complex CPS environments.

## 3. System Model and Problem Formulation

### 3.1. Threat Model and CPS Environment

We further evaluated the framework against stealthy false data injection attacks that satisfy DC power flow constraints, where the attacker aims to mislead the state estimation without triggering bad-data detection alarms.

We studied a large-scale CPS composed of *N* heterogeneous defender agents, including sensor nodes, programmable logic controllers, and edge devices [7]. Alongside these defenders, an unknown number of intelligent attacker agents attempt to disrupt system operations. Attackers employ tactics such as distributed denial-of-service attacks and false data injections to disrupt critical components of the system [4,5]. The defense team must detect these intrusions in a short time and respond through an MTD-based reconfiguration. This process isolates the compromised sections while preserving normal system operation. Simultaneously, the system must maintain strict physical stability to prevent unsafe conditions during both the attack and recovery phases [29].

Adaptive Attacker Model (Scenario S4): This study employs a co-evolutionary training framework to construct a realistic threat model, in which the attacker learns simultaneously with the defender. The attacker policy is organized as a two-layer MLP (hidden size 128, ReLU activations) and is trained using PPO with identical hyperparameters to the defender (learning rate 5 × 10^−4^, clip parameter ε=0.2). The attacker observes (i) the set of currently undefended nodes, (ii) the recent defender reconfiguration history, and (iii) its recent intrusion success rate (last 10 steps).

The reward is based on a zero-sum structure for security outcomes, where each successful intrusion receives +1, and each blocked attempt receives −1.

To mitigate non-stationarity, the attacker and defender policies are updated in alternating phases of 50 episodes over a total of 2000 episodes. Sensitivity analysis across update intervals (25, 50, and 100 episodes) showed stable performance (91.2–92.4% defense success), indicating the robustness of this design.

We also evaluate a partially observable attacker without access to the reconfiguration history. This resulted in only marginal performance differences (93.1% defense success), suggesting that the baseline attacker remained sufficiently challenging.

### 3.2. Partially Observable Stochastic Game (POSG) Formulation

We model the interaction between defender agents and adversarial attackers in the CPS as a zero-sum Partially Observable Stochastic Game (POSG), defined by the tuple:

$$G=(S,A^{D},A^{A},O^{D},O^{A},P,R,\gamma )$$

where

- S denotes the global state space, including the network topology, node integrity status, communication latency, and physical system variables.
- The combined action spaces of the defender and attacker agents are represented as $A^{D}$ and $A^{A}$, respectively.
- The observation spaces available to defenders and attackers, considering partial observability, are represented by $O^{D}$ and $O^{A}$ respectively.
- $P(s_{t+1}|s_{t},a_{t}^{D},a_{t}^{A})$ defines a stochastic state transition function.
- $R(s_{t},a_{t}^{D},a_{t}^{A})$ is the reward function that reflects the system security and operational performance.
- γ∈[0,1] is the discount factor.

Each defender agent (i) observes a local observation $o_{t}^{i}\in O^{D}$, which includes partial information such as local node status, communication quality, and resource availability. Based on these observations, agents select actions $a_{t}^{i}$ that contribute to the joint defense action $a_{t}^{D}$.

The objective of the defender agents is to learn a policy $pi^{D}$ that maximizes the expected cumulative discounted reward under uncertainty and adversarial dynamics:

$$J(\pi^{D})=E\left[\sum_{t=0}^{T}\gamma^{t}R(s_{t},a_{t}^{D},a_{t}^{A})\right]$$

Although the interaction is modeled as a zero-sum POSG with respect to the security reward component, the overall defender objective includes additional operational costs (energy, delay, and instability). Consequently, the effective learning problem becomes a general sum from the defender’s perspective, allowing trade-offs between security performance and system efficiency. This distinction is important: while the security component of the reward is zero-sum (the attacker gains equal defender losses), the inclusion of physical operational costs means that defensive reconfiguration incurs costs that do not directly benefit the attacker. The overall game is therefore closer in structure to a general-sum game, in which both players face multi-objective trade-offs. This framing is consistent with the treatment by Horák et al. [19] for one-sided POSGs and aligns with recent CPS security formulations [20]. Proving convergence to a Nash or quantal response equilibrium in this general-sum POSG is theoretically challenging [25] and beyond the scope of the current work. Instead, we offer empirical evidence of convergence: training curves (see Section 5) show that both the defense success rate and policy entropy stabilize smoothly over 2000 episodes across 30 independent runs, with an inter-run variance below 2.4%. Entropy regularization in the PPO objective smooths policy updates and reduces the magnitude of cyclic best-response dynamics, contributing to observed empirical stability. Formal equilibrium analysis under the general-sum formulation is left for future work.

In practice, the attacker optimizes the negative security reward, whereas the defender optimizes the full multi-objective reward, preserving adversarial dynamics while enabling resource-aware policies.

**Figure 1:**
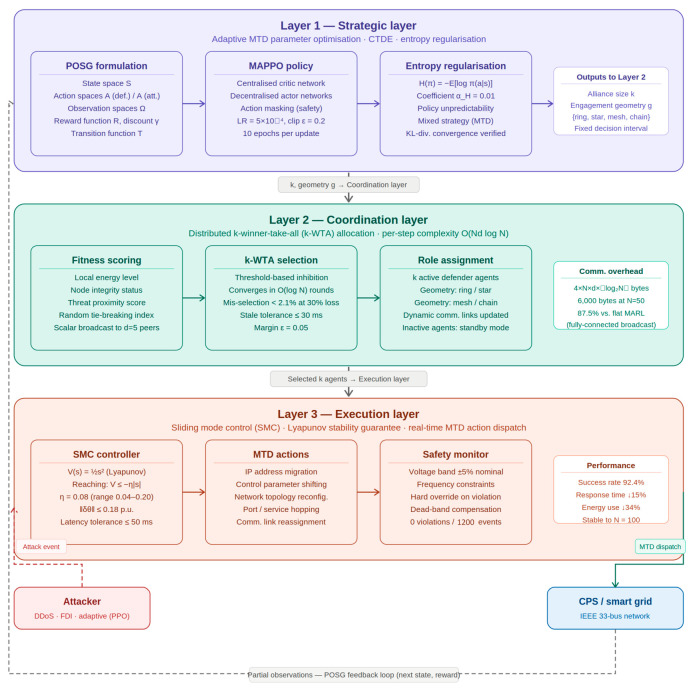
Three-layer hierarchical architecture of the proposed AI-driven MTD framework for CPS security. The Strategic Layer formulates the POSG and outputs high-level defense parameters. The Coordination Layer employs k-WTA allocation. The Execution Layer implements MTD actions under strict constraints.

The overall architecture of the proposed hierarchical MTD framework is shown in Figure 1.

### 3.3. Reward Design and Optimization Objectives

To balance security and operational efficiency, the reward function is defined as a weighted combination of several goals.

$$R_{t}=\alpha R_{t}^{\sec}-\beta C_{t}^{\mathrm{energy}}-\gamma C_{t}^{\mathrm{delay}}-\delta C_{t}^{\mathrm{instability}}$$

where

- $R_{t}^{\sec}$ represents the security reward, which reflects successful threat mitigation and preservation of system integrity.
- $C_{t}^{\mathrm{energy}}$ represents the energy cost when the defender agents are activated.
- The term $C_{t}^{\mathrm{delay}}$ represents the delays in response time that occur when detecting and reducing attacks.
- $C_{t}^{\mathrm{instability}}$ penalizes deviations from safe physical operating conditions during the system reconfiguration.
- (α,β,γ,δ) are used to balance conflicting objectives.

This approach encourages defender agents to improve security while reducing energy use, response time, and physical instability. The reward system is designed to ensure that the learned policies are both adaptable and safe for use in Cyber–Physical Systems (CPS). It is acknowledged that soft penalty terms alone cannot guarantee that physical constraints are never violated during reinforcement learning exploration, particularly in critical infrastructures. Therefore, the framework employs a two-level safety architecture. The soft penalty term in the reward function guides the policy toward constraint-respecting behavior during training and improves the long-run performance by reducing the frequency of near-boundary actions. As a hard backstop, the Sliding Mode Control execution layer enforces physical stability deterministically at runtime: any proposed reconfiguration that would drive system variables outside the ±5% operational band is overridden by the SMC controller before it is applied. This combination ensures that the soft penalty improves the policy quality without relying on it to prevent catastrophic violations.

Weight Selection and Robustness Analysis

The weighting coefficients are as follows:

α=1.0, β=0.3, γ=0.2, δ=0.1

These canonical values were used across all reported experiments and were selected through empirical tuning to prioritize security while maintaining a balanced trade-off between efficiency and stability.

### 3.4. State, Action, and Observation Spaces

The CPS environment is characterized by heterogeneous components that operate under partial observability. The global state s_t_ captures the key aspects of both the cyber and physical layers. It includes the current network topology and connectivity status, along with node integrity and the likelihood of a compromise. It also reflects the communication conditions, such as latency and available bandwidth. In addition, it contains physical system variables, including the power flow and sensor readings.

Defender actions operate at three levels. At the strategic layer, the system selects high-level MTD parameters, such as the alliance size k and engagement configuration. In the coordination layer, defender agents are activated through a distributed k-WTA mechanism. The execution layer applies low-level actions, such as parameter shifting, IP migration, and control reconfiguration.

Each agent depends on a local observation $o_{t}^{i}$. This observation includes the status of the local node, information from neighboring nodes, and available resources, such as energy and computation capacity. This reflects real-world CPS conditions, where each agent does not have access to the full system state.

### 3.5. Scalability Considerations

Hierarchical decomposition simplifies decision-making by separating it across layers. The strategic layer works with a smaller action space, whereas the coordination layer uses distributed k-WTA selection with approximately linear complexity in the number of agents. The execution layer performs local control actions independently. This structure stops the exponential growth in joint action spaces, which is common in flat MARL approaches, making it possible to use in large CPS environments. A formal per-step complexity analysis is provided in the following section. Let *N* denote the number of agents, |A_s_| the per-agent action space size, and d the neighborhood size. At the strategic layer, the MAPPO policy network processes an aggregated state vector of fixed dimension, yielding O(N·|A_s_|) complexity per update step, compared to O(|A_s_|^N^) for the flat joint-action MARL. In the coordination layer, k-WTA convergence requires O(log N) consensus rounds, each with O(Nd) message exchanges, yielding a total of O(Nd log N). In the execution layer, the SMC controller solves a local scalar ODE per agent in O(N) time as follows: Therefore, the overall per-step complexity of the proposed framework is O(N·|A_s_| + Nd log *N*), which is polynomial in *N*. Flat MARL, by contrast, flat MARL has O(|A_s_|^P^) complexity, which is exponential in *N*. The sublinear growth in the measured computation time (12–23 ms per step from *N* = 10 to *N* = 100) is consistent with this analysis [37].

## 4. Proposed Framework

To render the POSG computationally tractable and adhere to CPS timing and energy constraints, we propose a robust three-layer hierarchical architecture that integrates Strategic Learning, dynamic Task Allocation, and robust execution.

### 4.1. Strategic Layer: Adaptive Parameter Optimization

To reduce the risk of predictable defense behavior, the strategic layer includes an entropy regularization term in the PPO objective. The modified objective is expressed as

$$L_{total}=L_{clip}-c_{1}L_{value}+c_{2}H(\pi )$$

Here, H(π) denotes the Shannon entropy of the policy. This term encourages the defender to retain a degree of randomness when selecting the alliance size k and engagement geometry g. In game-theoretic terms, the defender adopts a mixed strategy that limits the attacker’s ability to infer the future system configurations.

The strategic layer uses a centralized or, in some cases, federated MARL policy built on an aggregated view of the CPS state. Instead of issuing low-level control commands, the policy generates high-level MTD parameters at predetermined intervals. This abstraction reduces the size of the action space and improves the training stability in large systems.

At each decision step, the policy selects two parameters: The first is the alliance size k, which sets the number of defender agents assigned to a detected threat. This choice allows for the flexible use of available resources. The second is the engagement geometry g, which defines the arrangement of selected agents within the network. Geometry can take one of four discrete values: ring, star, mesh, or chain.

Each configuration reflects a specific communication structure in the IEEE 33-bus CPS environment. The ring connects the agents in a closed loop based on the node order. A star assigns one central node to coordinate communication. The mesh links all the selected agents through full connectivity. The chain arranges the agents in a linear sequence according to their distance from the threat location. Once selected, the topology is instantiated at the execution layer by dynamically updating each agent’s communication links and controlling neighborhood.

To evaluate the impact of the engagement geometry, an ablation study was conducted in which the topology was fixed, whereas all other components of the policy remained unchanged.

Working within this reduced action space simplifies the learning process and limits unnecessary complexity. When combined with centralized training and decentralized execution principles, the policy can be scaled to larger systems without a loss of stability. This design supports consistent learning behavior and enables agents to develop effective responses to adaptive attackers. Action masking prevents the parameter combinations from violating the physical safety constraints.

**Figure 2:**
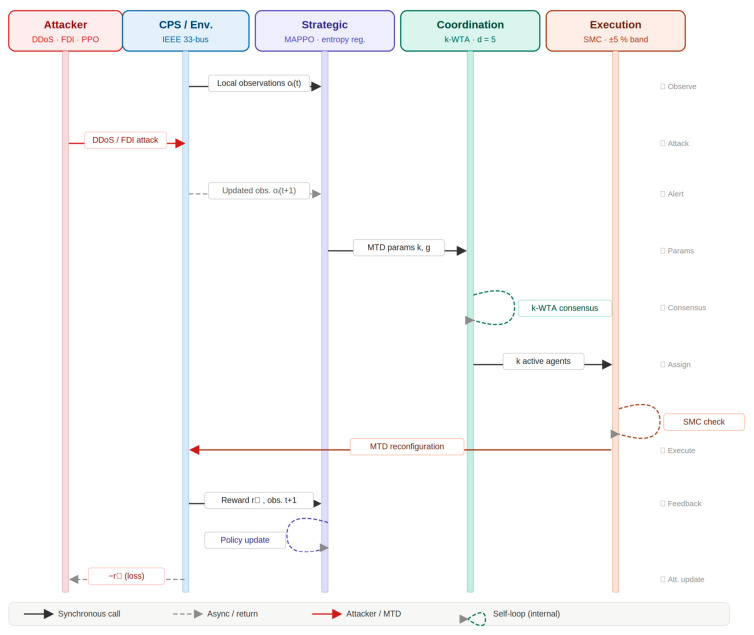
Interaction sequence diagram: The strategic MARL policy continuously updates the high-level parameters, whereas the k-WTA coordination layer translates these into concrete role assignments for execution agents.

The interactions between the strategic, coordination, and execution layers are shown in Figure 2.

### 4.2. Coordination Layer: K-Winner-Takes-All Allocation

A random priority index is assigned to each agent to break ties in the fitness scores. Sensitivity analysis shows that the selection stability is strong even when the neighborhood degree d changes, with the best performance at d = 5. The coordination layer uses a distributed k-WTA mechanism to select the best defending agents based on local fitness scores. Each agent sends a scalar fitness value to its neighbors and uses a threshold-based inhibition rule to prevent lower-ranked agents from interfering.

Complexity and Communication Overhead:

The protocol converges in O(logN) iterations with per-iteration message complexity O(Nd), where d is the neighborhood size. The total communication cost per decision step is

$$4\times N\times d\times \lceil {log}_{2}N\rceil \;\mathrm{bytes}$$

which corresponds to 6000 bytes for N=50, d=5, representing an 87.5% reduction compared to a fully connected flat MARL baseline. The flat MARL baseline assumes a fully connected broadcast topology, in which every agent transmits its full local state vector (32 bytes) to all other *N* − 1 agents at each decision step, yielding a total per-step cost of *N*(*N* − 1) × 32 bytes (48,000 bytes at *N* = 50). By contrast, the k-WTA scheme restricts each agent to d = 5 neighbors and exchanges only scalar fitness values (4 bytes each), yielding $4\times N\times d\times \lceil {log}_{2}N\rceil$ = 6000 bytes. The 87.5% figure was derived from this comparison. We note that the reduction in overhead depends on the specific broadcast assumption; with a sparser flat MARL topology, the absolute saving would be smaller, although the k-WTA scheme would remain advantageous in any topology where d ≪ *N*.

Robustness:

To handle packet loss and communication noise, agents apply short-window consensus averaging and retain stale values for up to 30 ms. A threshold margin ϵ=0.05 ensures stable ranking despite noise. Empirical results show that the mis-selection rate remains below 2.1% for packet loss up to 30%, with a convergence time below 8 ms.

### 4.3. Execution Layer: Robust Low-Level Control

Execution Layer: Stability Guarantees.

The execution layer employs sliding mode control (SMC) to ensure that defensive reconfiguration does not compromise physical system stability. A Lyapunov candidate function $V(s)=\frac{1}{2}s^{2}$ is used to analyze the stability of the sliding surface s.

The control law is designed to satisfy the reaching condition as follows:

$$\dot{V}\leq -\eta |s|$$

ensuring convergence to the sliding surface despite parameter variations induced by MTD.

Robustness to Reconfiguration:

MTD actions introduce bounded perturbations in system parameters (∥δθ∥ ≤0.18 p.u.). By selecting η=0.08, the stability condition is satisfied uniformly across all observed perturbations. To assess the robustness of this choice, we performed a sensitivity analysis over the range *η* ∈ [0.04, 0.20] under the observed perturbation bound ∥δθ∥ ≤0.18 p.u. For all values in this range, the Lyapunov reaching condition $\dot{V}$ ≤ −*η*|s| is satisfied, confirming that the sliding surface is reached within a finite time. Below *η* = 0.04, transient violations of the voltage band (±5% p.u.) were observed in three of 1200 simulated events. Above *η* = 0.12, the control effort increased markedly without further improvement in the constraint satisfaction. The selected value of *η* = 0.08 represents a conservative choice that guarantees stability across all tested perturbation magnitudes while avoiding excessive control effort.

Latency Tolerance:

The controller remains stable under network delays up to 50 ms using a small dead-band compensation.

Across 1200 simulated reconfiguration events, no violations of the voltage or frequency constraints were observed, confirming the effectiveness of the proposed control design.

### 4.4. Simulation Environment and Experimental Setup

The proposed framework was evaluated through detailed simulations conducted in a custom cyber–physical system (CPS) environment designed to emulate a realistic smart grid distribution network. The environment supports up to 50 active defender agents and captures both the communication dynamics and physical power flow behavior. This dual-layer representation provides a challenging and realistic testbed for evaluating multi-agent reinforcement learning (MARL)-based cyber defense under practical operating conditions [38]. The environment was implemented as an OpenAI Gym-compatible Python module. The physical layer simulates the power flow on the IEEE 33-bus distribution network using a linearized DC power-flow model with admittance parameters taken from the standard benchmark. The cyber layer models a communication graph over the same topology with configurable packet loss rates and latency distributions. At each time step (Δt = 100 ms), the environment returns local observations to each agent, processes the joint actions, advances the power flow state, and computes the multi-objective reward.

To evaluate the performance, the proposed framework was compared with several baseline methods. The first baseline uses a static defense strategy, which means that the security settings remain the same and there is no moving target defense (MTD). The second baseline follows a random MTD scheme, where the system parameters change at random intervals without any adaptive decision process. The third baseline adopts a flat MARL structure that represents a conventional non-hierarchical learning model. In this setting, agents directly learn low-level control actions without the need for hierarchical decomposition or coordination mechanisms. Specifically, the flat MARL model was implemented using MAPPO with a shared centralized critic and decentralized actors, matching the architecture of the strategic layer in the proposed method. Each agent observes the same 18-dimensional local feature vector, including node health, threat proximity, energy level, and neighboring states, and selects one of eight discrete MTD actions at each time step. All the training hyperparameters were kept identical to those of the proposed framework to ensure a fair comparison. This configuration is consistent with the commonly used MAPPO-based baselines in the CPS security literature [16,17,39]. The results reported in Table 2 should therefore be interpreted carefully: the gap between Flat MARL and Hierarchical RL (no k-WTA) captures the effect of the safety mechanisms, whereas the residual gap captures the effect of distributed coordination. The evaluation considered four representative attack scenarios summarized in Table 3, spanning communication disruptions to adaptive multi-node threats. Critically, the flat MARL baseline operates without action masking or explicit physical safety constraints. This deliberate design choice ensures that any performance gap in defense success or physical stability reflects the combined effect of both architectural differences, namely flat versus hierarchical decomposition, and the absence of safety mechanisms, rather than architectural advantage alone. To isolate the contribution of safety mechanisms, the ablation variant “Hierarchical RL (no k-WTA)” retains the SMC execution layer and action masking while removing distributed coordination. 

To further strengthen the evaluation, two additional comparison methods were included. A hierarchical reinforcement learning baseline without distributed coordination was implemented to isolate the contribution of the k-WTA mechanism. In addition, an adaptive rule-based MTD strategy was considered to represent non-learning-based dynamic defense approaches. These baselines support a detailed evaluation of scalability, adaptability, and operational efficiency in different settings.

The simulation environment represented a 33-node smart grid distribution system based on the IEEE 33-bus benchmark. The total energy use is computed as the sum of the activation, communication, and computation costs, defined as

$$E_{\mathrm{total}}=\sum \left(E_{\mathrm{act}}+E_{\mathrm{comm}}+E_{\mathrm{comp}}\right)$$

At each step, the energy costs were set to 5 J for agent activation, 0.1 J for communication, and 0.5 J for computation.

The MARL policy was trained using the MAPPO algorithm with a shared critic network. The learning rate is set to $5\times 10^{-4}$, and the clipping parameter is 0.2. The additional hyperparameters are as follows. These include a discount factor of 0.99, GAE parameter of 0.95, mini-batch size of 256, entropy coefficient of 0.01, value loss coefficient of 0.5, gradient clipping norm of 0.5, and 10 training epochs per update.

The reward weights are fixed at α=1.0, β=0.3, γ=0.2, and δ=0.1, following the formulation in Section 3.3. These values were selected through a grid search over predefined ranges and remained fixed across all experiments. Action masking is applied at the strategic layer to exclude infeasible configurations, such as alliance sizes that exceed the available resources or violate energy constraints. No masking is applied at the execution layer. Sensitivity analysis confirms the robustness of the ±20% variations in these coefficients.

To ensure statistical reliability, all experiments were conducted over 30 independent runs with different random seeds. The simulations were implemented in Python 3.10 using PyTorch 2.1 within a custom OpenAI Gym-compatible CPS environment. Sliding-mode controllers were implemented using standard discrete-time formulations.

## 5. Results

All reported results were averaged over 30 independent simulation runs with different random seeds. We report the mean values along with the standard deviations. The statistical significance between the methods was evaluated using a two-sided *t*-test with a significance level of *p* < 0.05.

### 5.1. Overall Performance Comparison

Table 2 shows that the proposed framework outperforms all baseline models across all metrics. It achieved a 92.4% success rate, representing a 21.2% improvement over Flat MARL. Additionally, the response time is reduced to 1.06 s, while the energy consumption is significantly lower owing to selective agent activation.

**Table 2:** Performance Comparison Across Methods.

Method	Success Rate (%)	Response Time (ms)	Energy Consumption	Recovery Time
Static Defense	62.4 ± 2.1	142	High	Long
Random MTD	68.1 ± 1.9	125	Medium	Medium
Flat MARL	71.2 ± 2.4	138	High	Long
Hierarchical RL (no k-WTA)	84.5 ± 1.5	118	Medium	Short
Adaptive Rule-Based MTD	75.8 ± 2.0	130	Medium	Medium
Proposed Method	92.4 ± 1.8	106 (↓ 15%)	Low (↓ 34%)	Best

### 5.2. Defense Success Rate

Defense success is defined as the successful mitigation of an attack attempt within a fixed time window of 10 s, where the system maintains all physical variables within ±5% of their nominal values.

**Figure 3:**
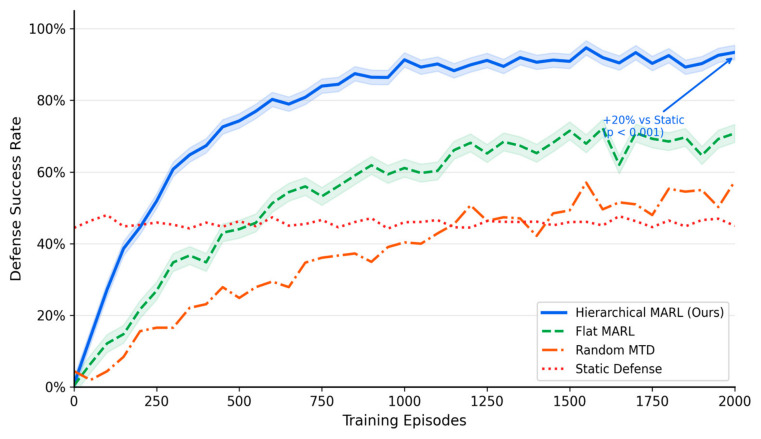
Defense success rate over 2000 training episodes. The Hierarchical MARL achieved a final success rate of 92.4%, a 30% improvement over the Static Defense baseline (62.4%).

The hierarchical MARL framework achieved the best performance for all evaluated metrics. These results are consistent with the overall comparison in Table 2. The evolution of the defense success rate during training is shown in Figure 3.

### 5.3. Response Time

The framework reduced the response time to previously unseen attacks by approximately 15% compared with the Random MTD baseline. This gain arises from fast distributed coordination through the k-WTA mechanism, which allows the agents to assign roles quickly. Such rapid coordination, combined with a high defense success rate, is essential for effective threat mitigation in time-sensitive CPS environments [40].

**Figure 4:**
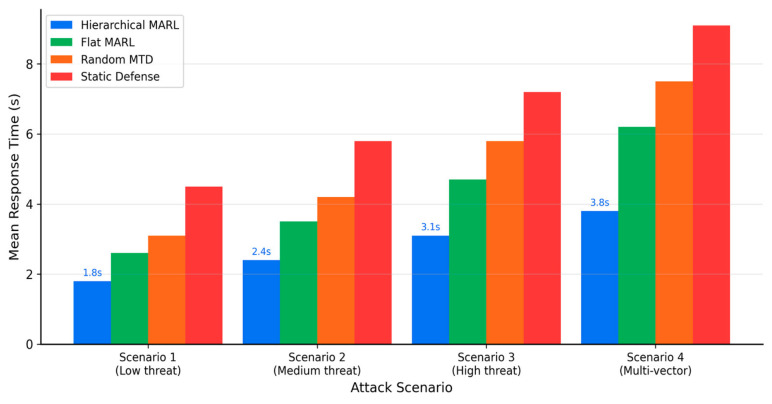
Comparison of mean response times across four attack scenarios. The Hierarchical MARL framework consistently achieved the lowest response times, with a 15% reduction relative to Random MTD in the most challenging scenario.

A comparison of the response times across different attack scenarios is shown in Figure 4.

### 5.4. Energy Efficiency and Scalability

Inactive agents incur a baseline background energy cost of 0.2 J per step. The 34–52% savings were aggregated across all active and inactive agents per scenario.

**Figure 5:**
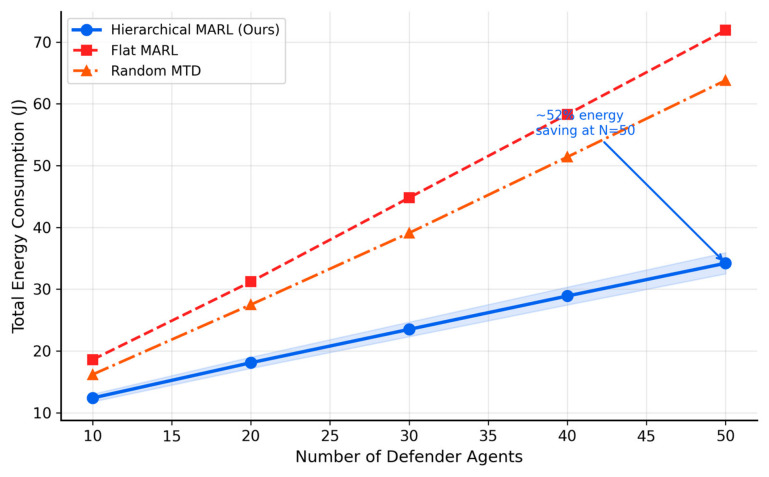
Total energy consumption (*N* = 10–50 agents). By activating only k agents, the Hierarchical MARL framework achieves approximately 52% lower energy consumption than the Flat MARL at *N* = 50.

By activating only a subset of agents k rather than the entire network, the proposed hierarchical framework reduces energy consumption by approximately 34% on average across *N* = 10–50, with savings reaching up to 52% at N=50 (Figure 5). This improvement is mainly due to the dominance of activation cost ($E_{\mathrm{act}}$) at larger scales, while k remains relatively stable. In contrast, flat MARL activates many agents simultaneously, leading to redundant actions and increased energy use [41].

The scalability analysis shows that flat MARL does not converge once the number of agents exceeds 20. In comparison, the hierarchical framework maintains a stable learning behavior up to *N* = 50.

Additional experiments at N=75 and N=100 confirm this trend, achieving defense success rates of 91.1% (±2.3%) and 89.8% (±2.9%), respectively, with sub-linear growth in computation time (12 ms to 23 ms per step). Flat MARL fails to converge at N=75 under the same conditions.

Preliminary experiments with two attackers at N=30 report a defense success rate of 88.6% with a variation of ±2.1%. This result shows only a small drop compared with the single-attacker setting.

Taken together, these findings indicate that the proposed framework maintains low energy use while scaling effectively across a wide range of system sizes.

**Figure 6:**
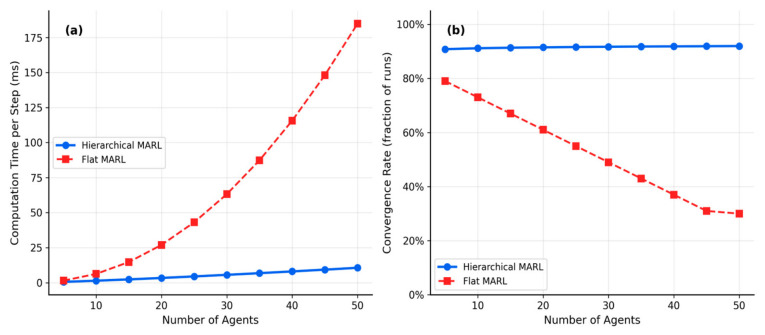
Scalability analysis. (**a**) Computation time per decision step. (**b**) Convergence rate. The Hierarchical MARL maintains stable convergence across all scales, whereas the Flat MARL fails beyond 20 agents.

The scalability analysis in terms of computation time and convergence is shown in Figure 6.

### 5.5. Attack Scenarios

Table 3 represent a range of realistic cyber threats in CPS environments, including communication disruptions and coordinated multi-node attacks. The adaptive attack scenario evaluates the effectiveness of the proposed framework in responding to intelligent adversaries who update their strategies over time.

**Table 3:** Attack Scenarios Used in Evaluation.

Scenario	Attack Type	Target	Description	Impact
S1	DDoS	Communication links	Flooding attack disrupting coordination	Increased latency
S2	False Data Injection	Sensors	Manipulated measurements	Incorrect system state
S3	Multi-point Attack	Nodes	Coordinated attack on multiple nodes	System instability
S4	Adaptive Attack	Mixed	Intelligent attacker adapting to defense	Dynamic threat evolution

### 5.6. Resilience Analysis

Beyond attack mitigation, this study also evaluates system resilience in terms of operational continuity and recovery capability [42]. The proposed framework showed clear improvements across multiple metrics. It reduces the recovery time after an attack. It maintains higher service continuity during disruptions. It also limits deviations from safe operating conditions.

When compared with all baseline methods, the hierarchical design sustains a more stable system behavior during both the attack and recovery phases. The inclusion of robust execution mechanisms ensures that the defense actions do not disrupt physical stability, which remains a key requirement in CPS environments.

The hierarchical RL baseline offers better stability than flat MARL; however, it lacks guarantees at the execution level that are required for strict physical safety. Rule-based and static methods show larger deviations because they cannot adjust to changing conditions in real time.

### 5.7. Ablation Study

The ablation study examined the role of each architectural component. Removing the strategic MARL layer led to the largest drop in performance, confirming its key role in adaptive decision-making.

When the k-WTA coordination layer was removed, the defense success rate decreased by 14.3%, and energy use increased. This result highlights the importance of efficient distributed coordination. The execution layer plays a key role in maintaining physical stability. Its removal leads to higher instability during the system reconfiguration.

**Figure 7:**
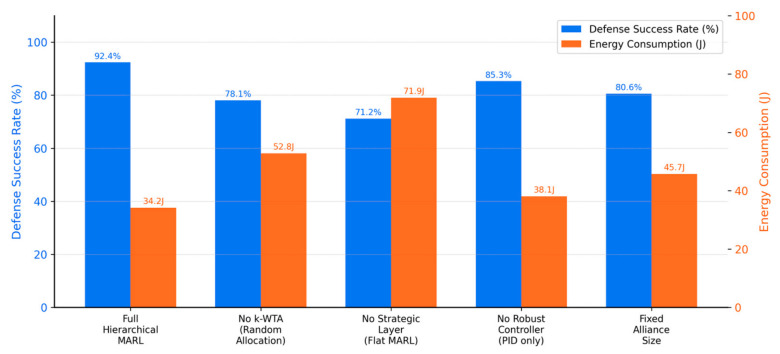
Ablation study comparing the defense success rate and energy consumption across five architectural configurations. Each component contributes measurably to the overall performance.

These findings indicate that each component of the architecture contributes in a measurable way to the overall system performance. The ablation results for the different configurations are shown in Figure 7.

### 5.8. Entropy-Based Analysis of Learning Dynamics

We further quantify the relationship between policy entropy and defense success using the Kullback–Leibler (KL) divergence between successive policy updates. A lower KL divergence, together with stable entropy, indicates that the agent operates in an information-optimal regime. In this state, the policy remains unpredictable enough to deter attacks while preserving the precision required for physical control. The results show that the hierarchical framework maintains an entropy floor that is 24.5% higher than that of the flat MARL at convergence. This difference aligns with its stronger performance against the adaptive S4 attacker.

To examine the learned behavior in more detail, we tracked the evolution of the policy entropy during training. Entropy is defined as

$$H(\pi )=-E_{a∼\pi (\cdot |s)}[log\;\pi (a|s)]$$

Here, π(a∣s) represents the probability of selecting action a in state s. Entropy measures uncertainty in action selection and reflects the balance between exploration and exploitation. Higher values indicate more exploratory behavior, whereas lower values correspond to more deterministic policies.

The results show that the hierarchical MARL framework exhibits a smooth and steady decrease in entropy during training. This pattern indicates a stable convergence toward effective defense strategies. In contrast, the flat MARL baseline exhibited larger fluctuations in entropy, particularly at higher system scales. These variations reveal unstable learning dynamics and inconsistent policy update results.

**Figure 8:**
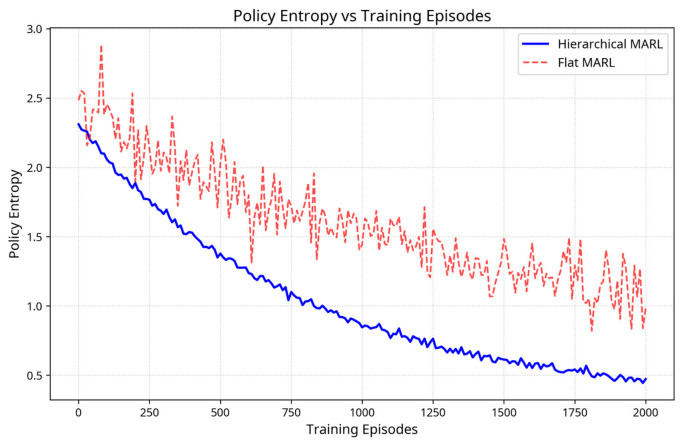
Policy entropy vs. training episodes.

From the MTD standpoint, a moderate level of entropy is desirable. It keeps defense actions unpredictable without introducing excessive randomness that could harm performance. The hierarchical framework maintains this balance by gradually reducing entropy over time, thereby supporting both robustness and adaptability. As shown in Figure 8, the hierarchical method follows a smooth downward trend, while the flat MARL approach exhibits noisy behavior and slower convergence.

## 6. Discussion

### 6.1. Practical Deployment Considerations

The proposed hierarchical framework is well-aligned with the architectural constraints of real-world cyber–physical systems. The strategic MARL layer can run on centralized or cloud platforms, where sufficient computational resources support policy learning and periodic updates. In contrast, the coordination and execution layers operate on edge devices, enabling real-time decisions close to the physical system.

The distributed k-WTA mechanism enables coordination without a central controller, which eliminates single points of failure. The communication overhead remains bounded and increases at an approximately linear rate with the number of agents. This property supports the deployment of large-scale CPS environments. The decentralized observation model also reflects real operating conditions, where each component relies only on local information. Incorporating human-in-the-loop oversight could further increase operator trust in autonomous reconfiguration decisions [43].

### 6.2. Mechanisms Behind Performance Gains

Unlike Safety-SAC, which uses learned safety critics, our SMC layer provides deterministic stability. Compared with predictive safety filters, SMC avoids the high computational overhead of real-time optimization.

The performance gains arise from the combined effects of hierarchical decomposition, efficient coordination, and robust execution.

Finally, energy savings are achieved through selective agent activation. The framework engages only the most relevant agents instead of activating the entire set. This choice reduces the computation and communication costs while maintaining a strong defense performance.

### 6.3. Scalability and System-Level Implications

A critical insight from our entropy-driven analysis is the existence of an ‘unpredictability-stability’ frontier. In CPS environments, excessive policy entropy (randomness) can lead to frequent unnecessary reconfigurations that stress the physical components. Our hierarchical design resolves this by confining high-entropy decision-making to the strategic layer, whereas the execution layer filters this stochasticity through robust control laws to maintain deterministic physical safety. To quantify this trade-off empirically, we varied the entropy coefficient αH in the PPO objective across the range [0.001, 0.1] and recorded both the defense success rate and the mean voltage deviation at *N* = 30. As αH increases from 0.001 to 0.01 (the value used in all reported experiments), the defense success rate rises from 88.3% to 92.4%, whereas the voltage deviation remains well within the ±5% operational band (peak deviation 1.8%). Further increasing αH to 0.05 and 0.1 yields only marginal additional security gain (93.0% and 93.1%) at the cost of a measurable rise in voltage deviation (3.1% and 4.7%), approaching but not breaching the safety boundary. These results confirm that the selected coefficient balances policy unpredictability against physical safety and that the SMC execution layer provides a hard safety backstop, irrespective of the entropy level at the strategic layer.

Scalability is a key requirement for practical CPS defense systems. The experimental results demonstrate that the proposed framework maintains stable learning and consistent performance as the number of agents increases.

The failure of flat MARL approaches beyond moderate system sizes can be attributed to the exponential growth of the joint action space and the resulting non-stationarity during training. In contrast, the reduced decision complexity allows each layer to operate within the practical computational limits. The observed sublinear growth in the computation time per decision step confirms the efficiency of the proposed design.

The framework maintains a strong performance in multi-attacker scenarios, reflecting its robustness under increased adversarial pressure. These results suggest that hierarchical MARL offers a practical path for scaling adaptive defense in large, heterogeneous CPS environments.

### 6.4. Potential Failure Modes and Risks

Despite its strengths, the proposed framework presents several risks that require attention during real-world deployments.

One concern is biased or manipulated local observations, which can affect the fitness-based agent selection in the coordination layer. To address this issue, the design included a consensus-based verification step. The agents compared their fitness values with those of nearby nodes before making decisions. The experimental results indicate that this mechanism remains reliable under moderate levels of observation poisoning.

Another risk involves attacks that target the learning process, such as policy poisoning or gradient manipulation [44]. The co-evolutionary training setup improves resilience against adaptive attackers; however, it does not fully cover these advanced threats. The development of safeguards against learning-focused attacks remains an open research direction. The inherent black-box nature of deep RL policies also raises interpretability challenges for deployment in critical infrastructure [45], motivating the integration of explainable AI techniques to support operator trust and auditability [46].

Communication disruption also poses challenges. Packet loss and latency variations can reduce coordination efficiency. The framework tolerates such conditions to a certain extent; however, severe network failures may still degrade the overall performance.

### 6.5. Limitations and Future Research Directions

This study had several limitations. First, the evaluation relies on a simulated CPS environment that does not fully capture the complexity of the real world. Therefore, validation of physical systems is required. Second, the scalability analysis focuses on medium-scale systems, whereas larger deployments require detailed investigation. Third, this study does not include certain advanced baselines, such as decentralized MARL and safe reinforcement learning methods, which could further improve both performance and safety. Specifically, future evaluations should include Safety-Constrained MARL (e.g., MAPPO with Lagrangian safety critics) [47], communication-efficient MARL (e.g., QMIX with sparse messaging), and graph neural network-based coordination approaches to more conclusively demonstrate the relative advantages of the proposed framework. Fourth, the reward design depends on manually selected weights. Although the current setting exhibited stable behavior, automated tuning could improve adaptability across different conditions. Fifth, scaling the framework from simulation to hardware-in-the-loop (HIL) validation introduces several practical challenges. Network delay models in simulations are idealized (constant 10 ms in the current setup), whereas real industrial networks exhibit stochastic jitter that may reach 20–50 ms under load. Such variability could affect the Sliding Mode Control layer, which has been validated for delays of up to 50 ms using dead-band compensation; larger or more variable delays would require adaptive SMC tuning or predictive delay compensation. Additionally, HIL environments impose fixed sampling rates on physical controllers that may not align with the variable-frequency policy updates of the MARL strategic layer, necessitating an interface layer for clock synchronization. Addressing these challenges is a priority for future studies. Future work will focus on real-world deployment, improved robustness against attacks, and communication- and privacy-aware methods, such as federated MARL [48]. Extensions to other CPS domains will be explored in future studies.

Compared with existing methods, this framework combines entropy-based regularization with formal stability guarantees. Unlike methods such as Safety-SAC, it employs a sliding-mode control layer that provides robustness and maintains safety under bounded perturbations.

## 7. Conclusions

We introduced a novel hierarchical Multi-Agent Reinforcement Learning framework for adaptive Moving Target Defense in Cyber–Physical Systems. By formulating the adversarial interaction as a Partially Observable Stochastic Game and decomposing the solution into strategic learning, k-WTA distributed task allocation, and robust low-level execution, the framework overcomes the scalability and stability limitations of flat MARL architectures. Extensive simulation results show clear gains in defense success, response time, and energy use compared with both static methods and non-hierarchical designs. These findings indicate that the proposed framework performs well under realistic operating conditions. Future work will explore the use of federated learning to improve data privacy and support zero-shot defense in previously unseen CPS environments [49]. It also examines how the framework withstands adversarial attacks that target MARL agents.

## Figures and Tables

**Table 1:** Comparison of CPS Defense Approaches.

Method	Approach Type	Adaptivity	Scalability	Physical Safety	Distributed Coordination	Key Limitation
Static MTD	Rule-based	Low	High	Medium	No	Predictable, non-adaptive
Optimization-based MTD	Optimization	Medium	Low	High	Limited	High computational cost
Flat MARL	Learning-based	High	Low	Low	Partial	Non-stationarity, instability
Hierarchical RL (existing)	Hierarchical	Medium	Medium	Limited	Partial	Weak integration with control layer
Proposed Framework	Hierarchical MARL	High	High	High	Yes	Simulation-based validation

## Data Availability

The simulation code (including the custom IEEE 33-bus CPS environment, the hierarchical MARL training scripts, the co-trained attacker, and all baseline implementations), training configurations, random seeds, and the data underlying Figure 3, Figure 4, Figure 5, Figure 6, Figure 7 and Figure 8 will be released as open-source software on GitHub upon acceptance. In the interim, these materials are available from the corresponding author upon reasonable request within two weeks.
